# Systemic exosomal siRNA delivery reduced alpha-synuclein aggregates in brains of transgenic mice

**DOI:** 10.1002/mds.25978

**Published:** 2014-08-11

**Authors:** J Mark Cooper, PB Oscar Wiklander, Joel Z Nordin, Raya Al-Shawi, Matthew J Wood, Mansi Vithlani, Anthony H V Schapira, J Paul Simons, Samir El-Andaloussi, Lydia Alvarez-Erviti

**Affiliations:** 1 Department of Clinical Neuroscience, Institute of Neurology, University College LondonLondon, United Kingdom; 2 Department of Laboratory Medicine, Karolinska InstitutetHuddinge, Sweden; 3 Centre for Biomedical Science, Division of Medicine, UCLLondon, United Kingdom; 4 Department of Physiology, Anatomy and Genetics, University of OxfordOxford, United Kingdom; 5 Wolfson Drug Discovery Unit, Centre for Amyloidosis and Acute Phase Proteins, UCLLondon, United Kingdom

**Keywords:** α-Syn, RVG-exosomes, siRNA, transgenic mice

## Abstract

Alpha-synuclein (α-Syn) aggregates are the main component of Lewy bodies, which are the characteristic pathological feature in Parkinson's disease (PD) brain. Evidence that α-Syn aggregation can be propagated between neurones has led to the suggestion that this mechanism is responsible for the stepwise progression of PD pathology. Decreasing α-Syn expression is predicted to attenuate this process and is thus an attractive approach to delay or halt PD progression. We have used α-Syn small interfering RNA (siRNA) to reduce total and aggregated α-Syn levels in mouse brains. To achieve widespread delivery of siRNAs to the brain we have peripherally injected modified exosomes expressing Ravies virus glycoprotein loaded with siRNA. Normal mice were analyzed 3 or 7 days after injection. To evaluate whether this approach can decrease α-Syn aggregates, we repeated the treatment using transgenic mice expressing the human phosphorylation-mimic S129D α-Syn, which exhibits aggregation. In normal mice we detected significantly reduced α-Syn messenger RNA (mRNA) and protein levels throughout the brain 3 and 7 days after treatment with RVG-exosomes loaded with siRNA to α-Syn. In S129D α-Syn transgenic mice we found a decreased α-Syn mRNA and protein levels throughout the brain 7 days after injection. This resulted in significant reductions in intraneuronal protein aggregates, including in dopaminergic neurones of the substantia nigra. This study highlights the therapeutic potential of RVG-exosome delivery of siRNA to delay and reverse brain α-Syn pathological conditions. © 2014 The Authors. *Movement* Disorders published by Wiley Periodicals, Inc. on behalf of International Parkinson and Movement Disorder Society.

## INTRODUCTION

Combined with the ubiquitous presence of alpha-synuclein (α-Syn) aggregates in Lewy bodies (LBs), the finding of mutations in and multiplications of the α-Syn gene in familial Parkinson's disease (PD) all point to the central role of α-Syn in PD pathogenesis.^1^ In 2003, Braak and colleagues^2^ described the staging of PD pathology based on the progression of α-Syn pathology from the brainstem to the cortex, consistent with the pathological propagation along specific neural pathways,^2^ although this is not a feature observed in all PD patients.^3^ Given the central role of α-Syn in PD pathology, strategies to decrease the expression of α-Syn in neurons are attractive approaches to delay PD progression. Although gene therapy is a powerful tool to down-regulate the expression of α-Syn,^4^ one of the major challenges for clinical application is the development of a safe and efficient vehicle to deliver the therapy to the brain. The brain is a particularly difficult target for drugs of all classes because the blood–brain barrier excludes most drugs with a size greater than 400 Da.^5^ Furthermore, delivery of the therapy throughout the brain is required because of the widespread nature of the α-Syn pathology in PD. Gene therapy approaches used in clinical trials have involved local delivery of viral vectors containing complementary DNA (cDNA) encoding for a specific protein.^6^ However, these are limited by the requirement for stereotactic surgery and delivery restricted to a limited anatomically defined brain region. Although transvascular gene therapy using liposomes has been used in animal models,^7^ immune activation mediates the rapid clearance of the vehicle and concomitant decrease in efficacy when liposomes are re-administered, limiting the duration of the therapy. To overcome these limitations, we have developed a system that uses systemic administration of modified exosomes for nucleic acid delivery to the brain.^8^

Exosomes are extracellular vesicles (40-120 nm) of endocytic origin released by numerous cell types that act as natural carriers of messenger RNA (mRNA), microRNA, and proteins between cells.^9^ We have previously developed modified exosomes that specifically target the brain by expressing a brain-targeting peptide (rabies virus glycoprotein peptide; RVG) in the extra-exosomal N-terminus of Lamp2b, a protein found abundantly in the membrane of exosomes derived from primary dendritic cells. Previously we demonstrated that intravenous injection of RVG-exosomes containing small interfering RNA (siRNAs) into normal mice led to the targeted silencing of Beta-secretase 1 or glyceraldehyde 3-phosphate dehydrogenase (GAPDH) expression in the brain highlighting its potential therapeutic value for neurological diseases^8^.

To evaluate the potential of this approach for PD treatment we have assessed the ability of RVG-exosomes loaded with siRNA-targeted for α-Syn to decrease α-Syn and its aggregates in the brains of mice.

## Methods

### Cell Culture and Exosome Purification

Murine dendritic cells harvested from bone marrow were cultured in complete medium.^8^ Cells were transfected after 4 days with 5 µg RVG-Lamp2b plasmid and 5 µL of TransIT LT1 transfection reagent (Mirus Bio). Cell culture medium was changed on day 7, cell culture supernatant harvested after 24 hours, and exosomes isolated by serial centrifugation. Exosomes were resuspended in 0.1 M ammonium acetate.

### Exosome Treatment of Mice

All the experiments were approved by the Swedish Local Board for Laboratory Animals and the UK Home Office. Twelve- to 14-week-old normal C57BL/6 mice and 20- to 22-week-old C57BL/6 transgenic mice expressing the human S129D α-Syn cDNA under the PrP promoter were used for experiments. One hundred fifty micrograms of α-Syn siRNA was electroporated into 150 µg siRNA RVG-exosomes, centrifuged and resuspended in 80 µL of 5% glucose immediately before tail vein injection. Animals were analyzed 3 or 7 days after injection.

### Alpha-Synuclein Analysis

Alpha-synuclein protein differential solubility and total levels were analyzed by western blotting.^10^ Alpha-synuclein mRNA levels were analyzed by quantitative polymerase chain reaction (qPCR).^10^ Presence of α-Syn aggregates was analyzed by ThioS and immunohistochemical staining.^11^

Detailed methods and statistical analyses can be found in the Supplementary Data.

## RESULTS

### In Vitro Validation of siRNAs Against Alpha-Synuclein

**FIG 1:**
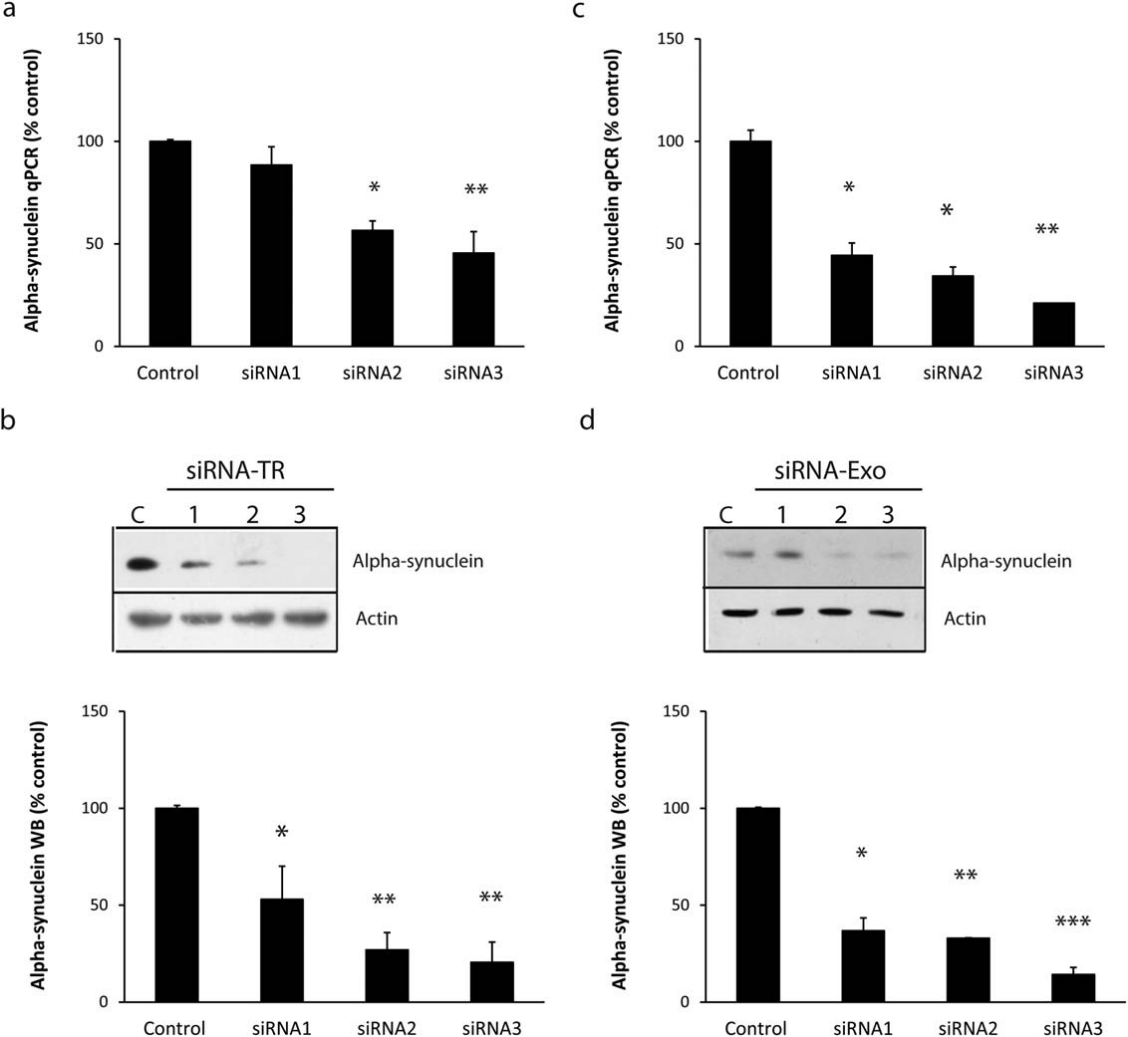
Analysis of α-Syn knockdown by various siRNAs in cell models SH-SY5Y cells over-expressing mouse α-Syn-HA were treated with 100 nM of three different siRNAs (siRNA 1, 2, or 3) using Hiperfect transfection reagent (A, B) or 3 μg RVG exosomes loaded with the different siRNAs (C, D). After 3 days, total α-Syn mRNA levels were quantified by qPCR relative to GAPDH (A, C) and mouse α-Syn (anti-HA antibody) protein levels relative to actin by Western blot (B, D). Data expressed as mean ± SEM (n = 4). Statistical analyses compared with control group, *p < 0.05, **< 0.01, ***< 0.001.

Three different siRNA sequences (siRNA1, 2, and 3) were designed to the coding sequence common to mouse and human α-Syn (Supplemental Data Table 1) to down-regulate their expression without targeting beta or gamma-synuclein (Supplemental Data Table 2). The ability of these siRNAs to knock down wild-type mouse α-Syn was assessed in human SH-SY5Y cells expressing mouse α-Syn-HA (mSyn-SHSY5Y). Using conventional lipofection reagents, we demonstrated a decrease in the total α-Syn mRNA in cells treated with each of the three siRNAs relative to controls (13%, 45%, and 55% decrease, respectively), although this was only statistically significant for siRNA2 and siRNA3 (Fig. 1A). The decrease in ectopic mouse α-Syn protein level was greater than the decrease in mRNA levels; significant decreases were obtained with all three siRNAs (45%, 70%, and 80% decrease, respectively) (Fig. 1B). The experiments were repeated using RVG-exosomes to deliver the siRNAs to the cells. RVG-exosomes were loaded with the three different siRNAs and added to the mSyn-SHSY5Y cell culture medium. After 72 hours, all three siRNAs delivered by RVG-exosomes reduced total α-Syn mRNA (55%, 65%, and 80% decrease, respectively), and protein (64%, 68%, and 85% decrease, respectively) levels (Fig. 1C, D). Small interfering RNA3 was selected as the most efficient sequence. These results validated the use of RVG-exosomes for siRNA delivery and suggested a higher efficiency of RVG-exosomes than a conventional lipofection agent (Supplemental Data Fig. 1).Thus, we next set out to investigate the feasibility of this approach to silence α-Syn in vivo.

### Down-Regulation of Endogenous Alpha-Synuclein in Normal Mouse Brain

The homogeneity of the RVG-exosomes used in this study was confirmed by nanoparticle tracking analysis demonstrating homogenous populations of exosomes with a size distribution peaking at a diameter around 100 nm (Supplemental Data Fig. 2). To investigate the brain distribution of RVG-exosomes after systemic delivery, RVG-exosomes labeled with a lipophilic IRDye (DiR) were injected intravenously into normal mice. After 24 hours the brains from mice treated with RVG-exosomes demonstrated a significant (3.8-fold) increase in fluorescence (*p* < 0.01) compared with untreated controls (Supplemental Data Fig. 2), which was throughout the brain, demonstrating a widespread distribution of RVG-exosomes.

**FIG 2:**
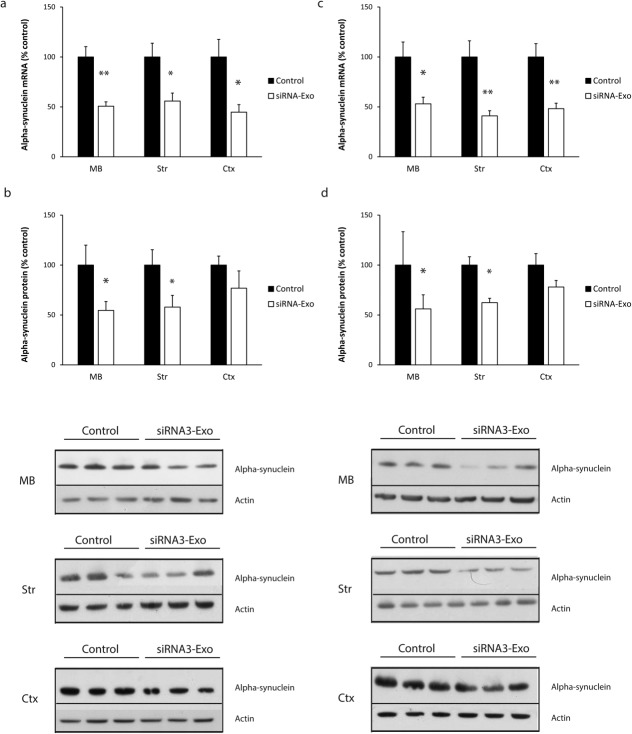
The effect of intravenous injection of siRNA3 RVG-exosomes on brain α-Syn levels in normal mice Normal mice were injected intravenously with siRNA3 RVG exosomes (150 μg) and the brain removed and dissected 3 days (A, B) and 7 days (C, D) after injection. Alpha-synuclein mRNA levels were analyzed by qPCR relative to GAPDH (A, C), and protein levels analyzed by Western blot relative to actin (B, D), in the midbrain (MB), striatum (Str), and cortex (Ctx). Typical Western blots showing α-Syn and actin blots for midbrain, striatum, and cortex samples from control and siRNA RVG-exosome treated mice are shown (B, D). Data normalized to control levels and expressed as mean ± SEM (n = 8). Statistical analyses compared with untreated control group, *p < 0.05, ** *p* < 0.01.

To examine the in vivo efficacy of siRNA3 RVG-exosomes to down-regulate endogenous α-Syn in the brain, RVG-exosomes loaded with siRNA3 were injected intravenously into normal mice. Brains were dissected 3 and 7 days after injection and analyzed for α-Syn mRNA and protein levels. After 3 days there were significant decreases in α-Syn mRNA levels in midbrain, striatum, and cortex samples (50%, 45%, and 55% decrease, respectively), with a concomitant decrease in α-Syn protein levels (45%, 43%, and 24% decrease, respectively) compared with untreated control mice (Fig. 2A, B). These decreases were similar 7 days post-injection, with mRNA levels in the midbrain, striatum, and cortex decreased by 47%, 59%, and 52%, respectively, and α-Syn protein levels decreased by 44%, 38%, and 22%, respectively (Fig. 2C, D). These results confirmed the in vivo potential for the systemic delivery of siRNAs by RVG-exosomes to decrease the expression of α-Syn in brain regions relevant to PD.

### Down-Regulation of Human Phospho-Mimic S129D Alpha-Synuclein in Transgenic Mouse

**FIG 3:**
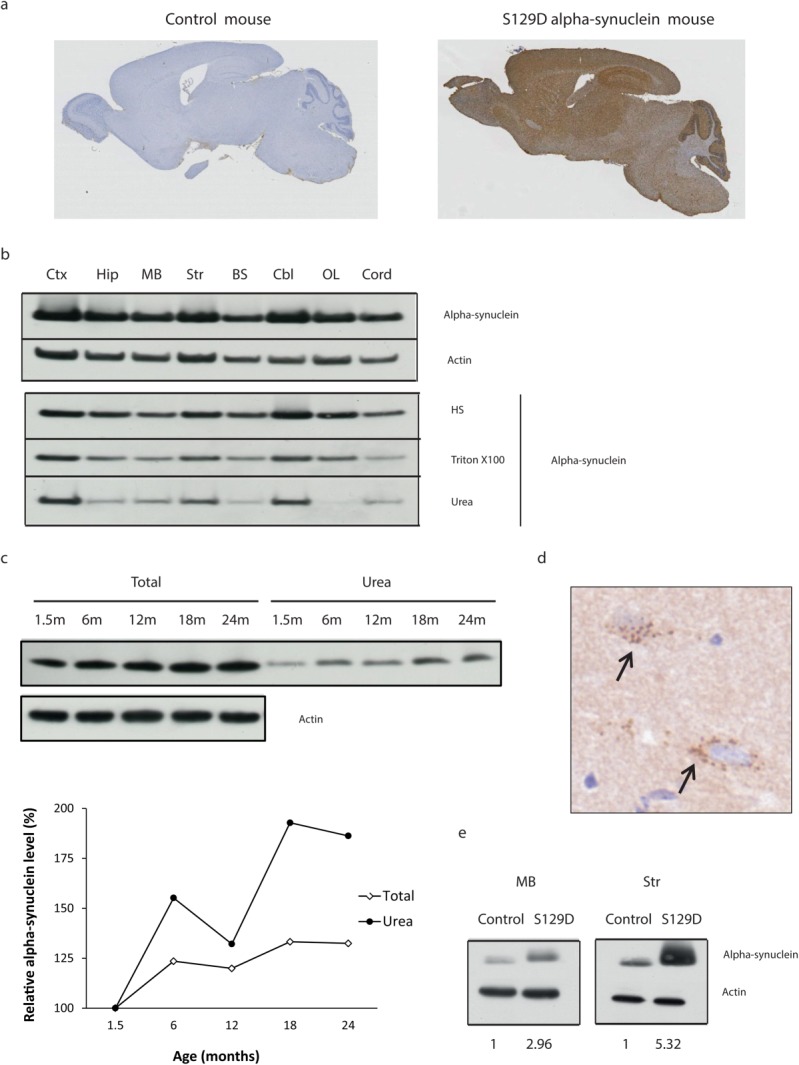
Characterisation of the features of the human S129D α-Syn transgenic mouse Tg13 (A) Immunohistochemical detection with DAB (brown staining) of S129D α-Syn HA expression using an anti-HA antibody in 6-month-old control (left panel) and Tg (right panel) mice. (B) Western blot analyses of whole tissue extract (upper panel) or sequential extractions with high salt (HS), Triton X-100, and urea/SDS (Urea) (lower panel) for cortex (Ctx), hippocampus (Hip), midbrain (MB), striatum (Str), brain stem (BS), cerebellum (Cbl), olfactory lobe (OL), and cord, using anti-HA or anti-actin antibodies. (C) Western blot analysis of whole midbrain extracts (Total) and triton X-100 insoluble material (urea) from Tg13 mice of different ages (1.5-24 months). Graph represents changes in total α-Syn relative to actin and changes in triton insoluble urea soluble α-Syn, data normalized to the values at 1.5 months. (D) Magnification of the anti-HA immunohistochemistry with DAB detection of the thalamus region of a Tg13 mouse at 5 months of age demonstrating cytoplasmic DAB-positive inclusions (arrows). (E) Western blot comparison of α-Syn expression in the midbrain and striatum of control and Tg13 (S129D) mice relative to actin, data normalized to the control values. [Color figure can be viewed in the online issue, which is available at wileyonlinelibrary.com.]

We have generated a transgenic (Tg) mouse model expressing the phospho-mimic human S129D α-Syn cDNA with a C-terminal HA tag under the control of the PrP promoter. This model (PrP-hSNCA-HA^S129D^ line 13; referred to as Tg13 below) demonstrates α-Syn expression throughout the brain (Fig. 3A, B). Triton X-100 insoluble α-Syn was evident from as young as 6 weeks of age and increased with age in all regions tested (midbrain shown Fig. 3C). Although all regions exhibited evidence of triton X-100 insoluble α-Syn, it appeared more prominent in the cortex, cerebellum, and striatum (Fig. 3B), consistent with increased aggregation associated with S129 phosphorylated α-Syn in LBs in PD.^12^ This is consistent with the immunohistochemical HA-positive cytoplasmic inclusions that were observed throughout the brain from 3 months of age (Fig. 3D). The level of S129D α-Syn expression relative to endogenous mouse expression was increased by between 3 to 5 times (Fig. 3E). However, the analysis of activity^13^ and motor skills^13^ at 6 months of age, or dopamine levels at 9 months, did not reveal any abnormality (Supplemental Data Fig. 3).

**FIG 4:**
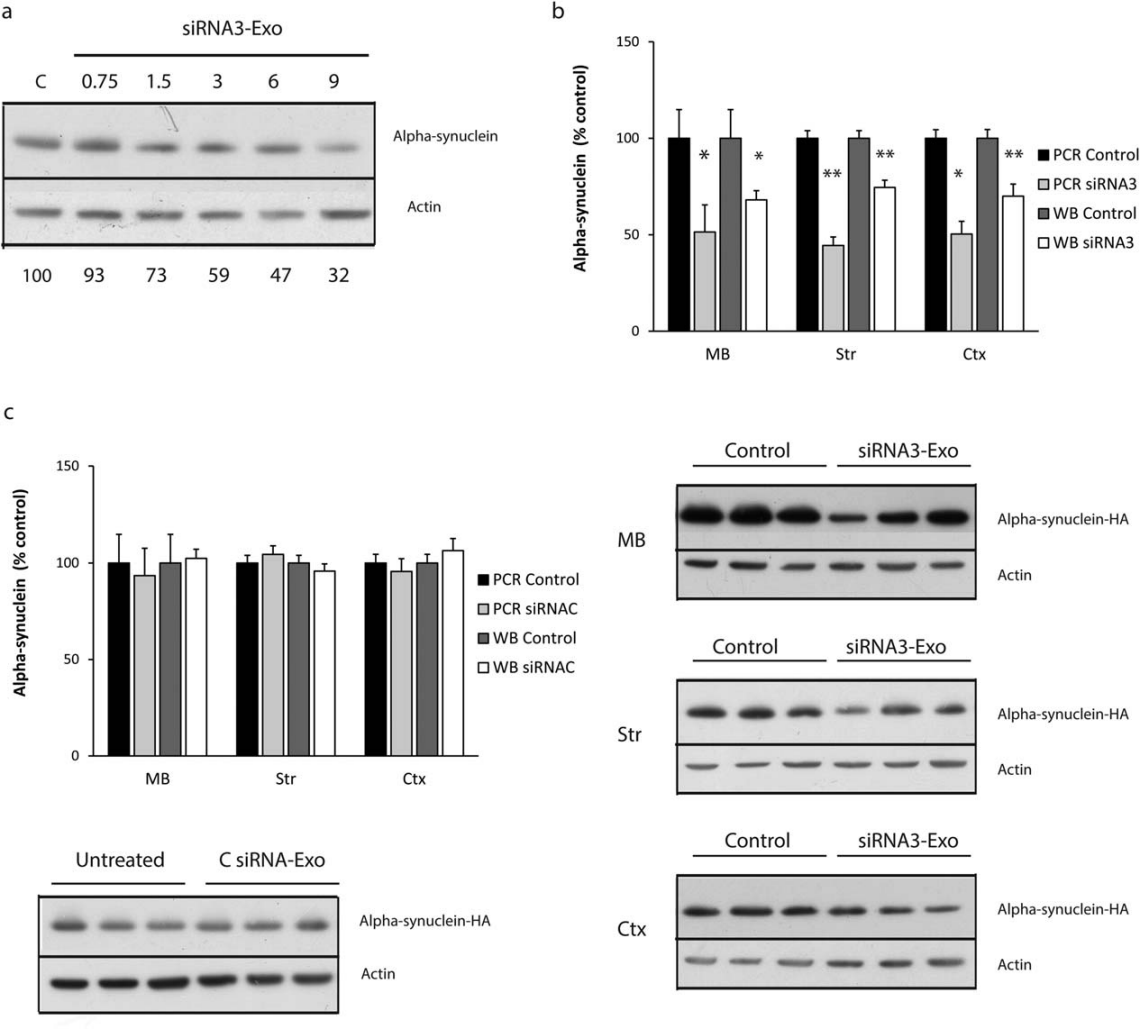
Down-regulation of human S129D α-Syn in SHSY5Y cells and Tg13 mice using siRNA3 RVG-exosomes (A) Western blot analysis of α-Syn-HA levels relative to actin 3 days after the addition of increasing amounts of siRNA3-RVG exosomes (0.75-9 µg exosome protein) to SH-SY5Y cells expressing human S129D α-Syn-HA; lower numbers represent α-Syn protein level relative to actin. (B) qPCR and Western blot analyses of human S129D α-Syn-HA expression in various brain regions from Tg13 mice (5 months of age) 7 days after intravenous injection with 150 μg siRNA3 RVG-exosomes (siRNA3), and compared with untreated controls (con). Mid brain (MB), striatum (Str), and cortex (Ctx) samples analyzed for S129D α-Syn-HA mRNA levels (PCR siRNA3) were expressed relative to GAPDH mRNA and S129D α-Syn-HA protein levels (anti-HA antibody) expressed relative to actin levels (WB siRNA3). Data were normalized to brain regions from matched controls (PCR control, WB control). Typical blots of the midbrain, striatum, and cortex samples are shown. Data expressed as mean ± SEM (n = 8). Statistical analyses compared with control group, *p < 0.05, **p < 0.01. (bold>C) As a control, Tg13 mice were injected with 150 μg CsiRNA RVG-exosomes (loaded with the control siRNA) and compared with untreated controls (control). Seven days after injection, brains were dissected (n = 8 per region) and analyzed as described in section B. A Western blot of the midbrain samples is shown.

Increased α-Syn aggregation is a characteristic pathological feature of PD in many brain regions; therefore, it is important to determine whether systemically delivered siRNA RVG-exosomes can influence α-Syn pathology in these regions. To confirm that siRNA3 is effective at decreasing the expression of human S129D α-Syn, we treated SH-SY5Y cells overexpressing human S129D α-Syn-HA with different concentrations of siRNA-RVG exosomes. There was a clear dose-dependent decrease in S129D α-Syn-HA protein relative to actin (Fig. 4A), confirming that siRNA3 RVG-exosomes can effectively down-regulate human S129D α-Syn.

Tg13 mice were injected intravenously with RVG-exosomes loaded with siRNA3 at 5 months of age or left untreated. After 7 days, α-Syn-HA mRNA and protein levels were analyzed in three and eight different dissected brain regions, respectively. The mRNA levels were significantly decreased in the three brain regions most relevant to PD; midbrain (49% decrease), striatum (56% decrease), and cortex (50% decrease) (Fig. 4B). This was paralleled by a significant decrease in S129D α-Syn-HA protein levels in the midbrain (32% decrease), striatum (26% decrease), and cortex (30% decrease), although the decrease was slightly less marked than that seen for the mRNA (Fig. 3B). Mean S129D α-Syn-HA protein levels were also down-regulated in the other five regions analyzed, this was significant for the cerebellum (decreased 45%), brain stem (decreased 29%), thalamus (decreased 44%), and spinal cord (decreased 30%), although these did not reach statistical significance for the olfactory lobe (decreased 16%) (Supplemental Data Fig. 4A, B).

**FIG 5:**
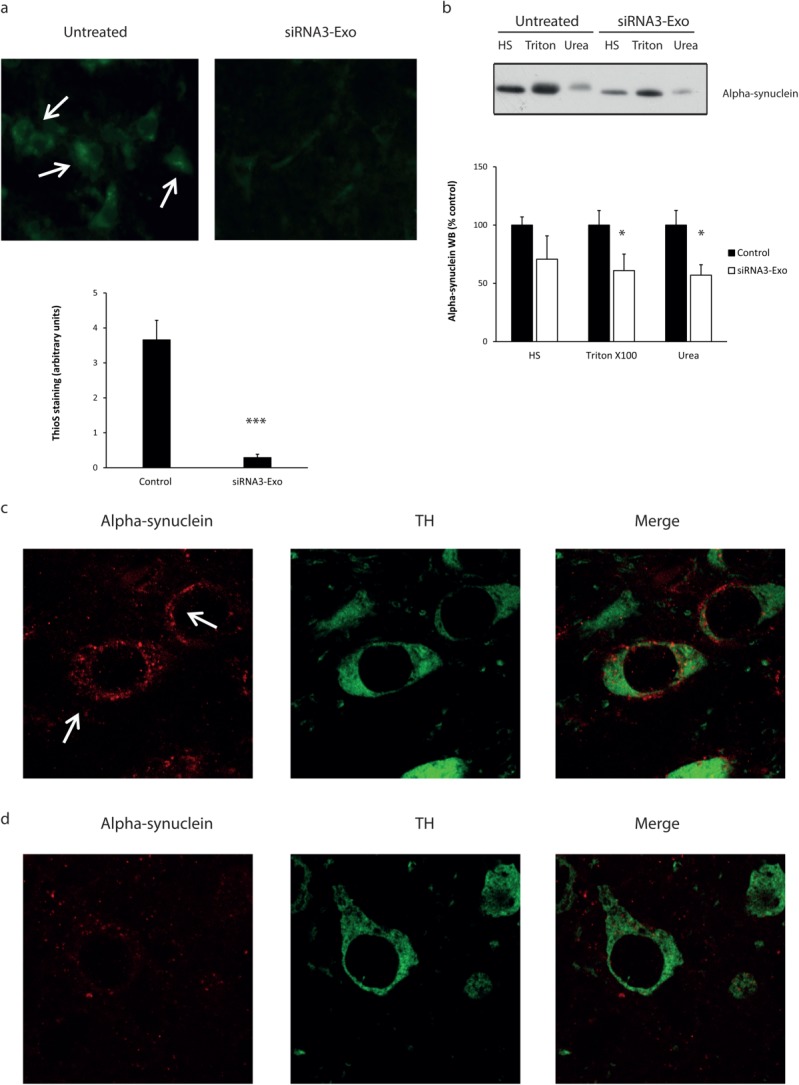
Assessment of α-Syn aggregates in Tg13 mice The influence of siRNA3 RVG-exosome treatment on α-Syn aggregates were assessed by (A) ThioS staining of aggregates in the mid brain of untreated and siRNA3 RVG-exosome treated Tg13 mice. ThioS-positive inclusions were clearly observed in the untreated mice (arrows); the ThioS staining was markedly decreased in the siRNA3 RVG-exosomes injected mice (siRNA3-Exo) brains. The ThioS staining intensity was quantified using Image J (20 neurones analyzed per animal). (bold>B) Sequential extraction of mid brain samples (3 mg) with high salt (HS), HS/Triton X-100 (Triton), and SDS/urea (urea). The gel was loaded with 5%, 5%, and 20% of each extract, respectively, and the blot probed with an anti-HA antibody. Data are expressed as mean ± SEM. Statistical analyses compared with control group, *p < 0.05, ***p < 0.001. Immunofluorescent analysis of midbrain sections from untreated (C) or siRNA3 RVG-exosome injected (bold>D) mice using antibodies to S129D α-Syn-HA (red, anti-HA) and tyrosine hydroxylase (green). [Color figure can be viewed in the online issue, which is available at wileyonlinelibrary.com.]

To confirm the specificity of the down-regulation, we tested a chemically modified siRNA sequence (control, CsiRNA) that differed from siRNA3 in one nucleotide (Supplemental Data Table 1). This control siRNA (CsiRNA) did not induce α-Syn down-regulation at mRNA (Supplemental Data Fig. 5A) or protein levels (Supplemantal Data Fig. 5B) in cell cultures. A new batch of Tg13 mice were injected with RVG-exosomes loaded with CsiRNA or left untreated. After 7 days there was no difference in α-Syn-HA mRNA or protein levels between CsiRNA RVG-exosome and untreated groups for the three brain regions tested (Fig. 4C), confirming the role of the siRNA3 in the α-Syn down-regulation observed with the RVG-exosome treatment.

To examine the influence of α-Syn down-regulation on α-Syn aggregation in the transgenic mice, midbrain sections were stained with the green fluorescent dye Thioflavin S (ThioS), which stains amyloid deposits. Untreated Tg13 midbrain sections demonstrated widespread cytoplasmic ThioS positivity (Fig. 5A, arrows); this is consistent with the presence of α-Syn aggregates and the increase in triton insoluble α-Syn in the brains of these mice (Fig. 3B). ThioS staining of the midbrain sections from the mice was greatly diminished 7 days after siRNA3 RVG-exosome treatment (Fig. 5A), with staining decreased by 84% (ImageJ quantification) relative to untreated samples (Fig. 5A). In midbrain samples from untreated mice, most of the S129D α-Syn was extracted in the high salt and triton fractions with a small proportion in the sodium dodecyl sulfate (SDS}/urea fraction corresponding to insoluble aggregated protein (Fig. 5B). After treatment of Tg13 mice with siRNA3 RVG-exosomes sequential extraction of midbrain samples showed a decrease in α-Syn levels in all 3 fractions with slightly greater reductions in the membrane-associated (Triton X-100) and insoluble (SDS/urea) fractions (Fig. 5B), consistent with a decrease in both the soluble and aggregated forms of the protein. Finally, we assessed the immunohistochemical distribution of S129D α-Syn-HA in the pathologically relevant dopaminergic neurones in the substantia nigra pars compacta. In untreated transgenic mice α-Syn-HA puncta can be seen in tyrosine hydroxylase–positive neurones (Fig. 5C, arrow), which were decreased in the mice 7 days after injection with siRNAs RVG-exosomes (Fig. 5D, Supplemental Data Fig. 5C)

## Discussion

Although effective symptomatic treatments for PD are available and patient management has improved dramatically in recent years, PD is inexorably progressive, and there is currently no disease-modifying therapy.

Whereas the mechanisms underlying the clinical and pathological features of PD remain to be defined, α-Syn clearly plays a central role in the disease process with elevated α-Syn levels apparently sufficient to lead to some familial forms of PD.^14^,^15^ This suggests that the down-regulation of α-Syn is an attractive strategy to delay disease progression, even though it is not clear whether oligomers, protofibrils, or larger aggregates are the pathological form of the protein.^12^,^16^ A previous study demonstrated that infusion of modified siRNA against α-Syn into the hippocampus of normal mice down-regulated α-Syn in the hippocampus over 14 days.^4^ However, the highly invasive nature of the delivery method used and the confined distribution of the siRNA renders this approach to siRNA delivery unsuitable for therapy for diseases such as PD. In contrast, RVG-modified exosomes administered by peripheral intravenous injection are able to deliver siRNAs into central nervous system neurons,^8^ suggesting that siRNA knockdown of brain α-Syn may be practicable by this approach. Toward this end, we have optimized the siRNAs targeting of both mouse and human α-Syn to evaluate silencing of endogenous and ectopic expression in cell cultures.

The localization of RVG-exosomes labeled with the lipophilic IRDye to the mouse brain within 24h of intravenous injection confirmed the ability of these modified exosomes to distribute throughout the brain. This adds to our previous observation that fluorescent tagged siRNAs in RVG-exosomes were found in the brain, and GAPDH and BACE-1 levels were decreased in several brain regions after the injection of the appropriate siRNA-loaded RVG-exosomes.^8^ Using the same in vivo treatment strategy, we have now demonstrated that injection of α-Syn siRNA3 loaded RVG-exosomes caused a significant decrease in murine α-Syn mRNA and protein levels in the brain regions analyzed. The levels of down-regulation of both α-Syn mRNA and protein were similar 3 and 7 days after injection and comparable in both the midbrain and striatum, and consistent with our previous data targeting GAPDH.^8^ It is unclear why cortical α-Syn protein levels did not decrease as much despite similar knockdown of the mRNA levels with the other regions, this had not been observed with GAPDH knockdown and may indicate that cortical α-Syn may have a slower turnover than in other regions. Over this short period the mice did not demonstrate any clear clinical abnormality which is in keeping with α-Syn knock-out mice, which are relatively normal, although knockout mice presented with altered dopamine release and striatal dopamine reduction.^17^ Although the RVG-exosome-siRNA therapy does not lead to a complete loss of α-Syn, prolonged depletion in adulthood may be functionally more dramatic, so it will be important for future studies to perform detailed clinical evaluations in mice with prolonged α-Syn down-regulation.

Lewy bodies are a key pathological feature of the disease and contain large amounts of α-Syn aggregates, and although their role in the disease mechanisms is not clear, the injection of preformed α-Syn fibrils^18^ or human LB^19^ can propagate some of the pathological features in mice. Consequently we needed to determine whether siRNA-loaded RVG-exosomal therapy can impact on the level of α-Syn aggregates. Alpha-synuclein aggregates are not a common feature of transgenic mouse models overexpressing α-Syn.^20^ However, α-Syn in Lewy bodies is highly phosphorylated at serine 129^21^ and S129 phosphorylated α-Syn is more prone to aggregate.^21^ Consequently we have generated a mouse model based on the overexpression of the human phospho-mimic S129D α-Syn under the PrP promoter. In this model α-Syn aggregates were apparent throughout the brain from 3 months of age, including in TH-positive neurons of the substantia nigra. This is in keeping with the expression of S129D α-Syn by Adeno-associated virus in mouse brains,^22^ which pointed to S129 phosphorylation as an important factor in α-Syn deposition in cellular inclusions.^23^

As a proof of principle, we treated 5-month-old human S129D α-Syn transgenic mice with siRNA3 loaded RG-exosomes to identify whether the treatment had any impact on the α-Syn aggregates seen in these mice. Our results confirmed that systemic siRNA RVG-exosome treatment decreased the total levels of human S129D α-Syn protein throughout the brain, including the midbrain, cortex, and striatum, which are important areas affected by α-Syn deposition at different stages of PD pathology. This was not observed using a modified siRNA sequence, demonstrating that the down-regulation observed in the treated mice was attributable to the efficient intracellular delivery of the active siRNA. Comparison between the siRNA3 RVG-exosome treatments of normal mice with the S129D transgenic mice revealed a similar reduction in the α-Syn mRNA levels relative to their respective untreated controls but slightly less effective in decreasing the S129D α-Syn protein levels. Although this may relate to the higher total α-Syn levels in the transgenic mice, it is more likely to reflect the presence of aggregated protein, which may be more inert and require longer gene down-regulation to have a greater impact. It is particularly notable that the differential solubility of α-Syn indicated that both soluble and aggregated α-Syn was decreased by the therapy but there remained some triton-insoluble α-Syn after 7 days' therapy. The decrease in α-Syn aggregates was further supported by the loss of thioflavin S-positivity in the midbrain after the therapy. The dynamics of intra-cellular α-Syn aggregates are not known. However, there is evidence from several mouse models of Huntington's disease that the level of huntingtin aggregates decrease within days of the levels of huntingtin protein being decreased either by turning off expression^24^ or by using siRNA to decrease expression.^25^ This suggests that in transgenic mouse models at least htt aggregates are not fixed but are being generated and either degraded or dispersed and that a decrease in htt levels is sufficient to decrease the aggregate levels. Our data suggest this is also true for α-Syn aggregates.

In conclusion, using systemic administration of siRNAs, we have been able to significantly decrease the level of endogenous mouse α-Syn, and a pro-aggregating human form of α-Syn in a transgenic mouse model, in brain regions pathologically affected in Parkinson's disease. This is the first time this specific approach has been used successfully in vivo to decrease the level of a human protein and more specifically show a decrease in the level of aggregated protein. These results reinforce the idea that this approach can be further developed to abrogate the disease progression in PD. Consequently it paves the way for this technology to be further developed for clinical trials for PD and opens a new avenue for treatment of a wide variety of neurological diseases, including Alzheimer's disease, Huntington's disease, and prion disease.

## Author Roles

1. Research Project: A. Conception, B. Organization, C. Execution; 2. Statistical Analysis: A. Design, B. Execution, C. Review and Critique; 3. Manuscript Preparation: A. Writing the First Draft, B. Review and Critique.

J.M.C.: 1A, 1B, 1C, 2C, 3B

P.B.O.W.: 1C, 3B

J.Z.N.: 1C, 3B

R.A.S.: 1C, 3B

M.J.W.: 3B

M.V.: 1C

A.H.V.S.: 3B

J.P.S.: 3B

S.E.A.: 1A, 1C, 2C, 3B

L.A.-E.: 1A, 1B, 1C, 2A, 2B, 2C, 3A, 3B

## Financial Disclosures

Cooper: Stock Ownership in medically-related fields none. Consultancies none. Advisory Boards none. Partnerships none. Honoraria none. Grants Parkinson's UK, Wellcome Trust / MRC joint call in neurodegeneration award. Intellectual Property Rights none. Expert Testimony none. Employment University College London. Contracts none. Royalties none. Other none.

AL-Shawi; Stock Ownership in medically-related fields none. Consultancies none. Advisory Boards none. Partnerships none. Honoraria none. Grants Alzheimer's Research UK; Royal Free London NHS Foundation Trust; National Institute for Health Research. Intellectual Property Rights none. Expert Testimony none. Employment University College London. Contracts none. Royalties none. Other none.

Simons: Stock Ownership in medically-related fields none. Consultancies none. Advisory Boards none. Partnerships none. Honoraria none. Grants Medical Research Council, British Heart Foundation, Alzheimer's Research UK, Royal Free (London) NHS foundation trust, National Institute for Health Research. Intellectual Property Rights 1 patent applied for; assigned to University College London. Unrelated to current ms. Expert Testimony none. Employment University College London. Contracts none. Royalties none. Other none.

Schapira: Stock Ownership in medically-related fields none. Consultancies honoraria have been undertaken or received from the following pharmaceutical companies: BI, Lundbeck, Zambon. Grants Parkinson's UK, Wellcome Trust / MRC joint call in neurodegeneration award, CoEN. Intellectual Property Rights none. Expert Testimony none. Employment University College London. Contracts none. Royalties have been received from a variety of publishing houses including Elsevier, Oxford University Press. Other none.

Alvarez-Erviti:. Consultancies none. Advisory Boards none. Partnerships none. Honoraria none. Grants Parkinson's UK. Intellectual Property Rights 2 patent applied for; assigned to Isis Innovation Limited. Expert Testimony none. Employment University College London. Contracts none. Royalties none. Other none.
