# Supplementary material for: Systemic exosomal siRNA delivery reduced alpha-synuclein aggregates in brains of transgenic mice
Source: Mov Disord. 2014 Aug 11;29(12):1476–85. doi: 10.1002/mds.25978 (PMC4204174; doi:10.1002/mds.25978)
Supplement: Supplementary file 6 [file mds0029-1476-SD6.docx]

| NAME | SEQUENCE |
| --- | --- |
| siRNA 1 | CAAAGAGCAAGUGACAAAdTdT |
| siRNA 2 | UGAGAAGACCAAAGAGCAAdTdT |
| siRNA 3 | GACAAAUGUUGGAGGAGCAdTdT |
| Control siRNA | mGmArCmArAmArUmGrUmUrGmGrAmGrGmArGmCrA |
| A-Syn F | GCCAAGGAGGGAGTTGTGGCTGC |
| A-Syn R | CTGTTGCCACACCATGCACCACTCC |
| HA F | GCCCCACAGGAAGGAATTCTGGA |
| HA R | AGCGTAGTCTGGGACGTCGTATG |
| Human GAPDH F | GAAGGTGAAGGTCGGAGT |
| Human GAPDH R | GAAGATGGTGATGGGATTTC |
| Mouse GAPDH F | HK-SY-mo-600 against murine GAPDH (PrimerDesign) |
| Mouse GAPDH R | HK-SY-mo-600 against murine GAPDH (PrimerDesign) |
