# Supplementary material for: Systemic exosomal siRNA delivery reduced alpha-synuclein aggregates in brains of transgenic mice
Source: Mov Disord. 2014 Aug 11;29(12):1476–85. doi: 10.1002/mds.25978 (PMC4204174; doi:10.1002/mds.25978)
Supplement: Supplementary file 7 [file mds0029-1476-SD7.docx]

**CCAAAGAGCAAGUGACAAA**

**GACAAAUGUUGGAGGAGCAC**

**UGAGAAGACCAAAGAGCAA**

**GGTGTGGCAACAGTGGCTGAGAAGACCAAAGAGCAAGTGACAAATGTTGGAGGAGCAGTGGTGACGGGTGTGAC ALPHA-SYNUCLEIN**

**51 G--V--A--T--V--A--E--K--T--K--E--Q--V--T--N--V--G--G--A--V--V--T--G--V--T-**

**GGTGTGGCTTCAGTGGCTGAAAAAACCAAGGAACAGGCCTCACATCTGGGAGGAGCTGTGTTCTCTGGGGCAGG BETA-SYNUCLEIN**

**51 G--V--A--S--V--A--E--K--T--K--E--Q--A--S--H--L--G--G--A--V--F--S--G--A--G-**

**GCGTGACCTCAGGCCTGCCTTGGGGCTGGGGCTGGGGTGGAGGCCAGCCAGTGTCCTCCCATAGTGGCCGAGAA GAMMA-SYNUCLEIN**

**51 S--V--T--S--G--L--P--W--G--W--G--W--G--G--G--Q--P--V--S--S--H--S--G--R--E-**

**GACAAAUGUUGGAGGAGCAC siRNA3**

**UGAGAAGACCAAAGAGCAA siRNA2**

**CCAAAGAGCAAGUGACAAA siRNA1**
