# Supplementary material for: Systemic exosomal siRNA delivery reduced alpha-synuclein aggregates in brains of transgenic mice
Source: Mov Disord. 2014 Aug 11;29(12):1476–85. doi: 10.1002/mds.25978 (PMC4204174; doi:10.1002/mds.25978)
Supplement: Supplementary file 8 [file mds0029-1476-SD8.docx]

**SUPPLEMENTARY METHODS**

Generation of transgenic mice

Transgenic mice were generated as previously described^1^. Briefly, the human S129D alpha-synuclein cDNA with a C-terminal HA tag was cloned into the pPrP vector^2^ containing the promoter and exons 1 and 2 of the mouse prion protein gene. The transgene fragment was isolated from the plasmid vector and microinjected into pronuclei of one-cell eggs obtained from C57BL/6 × CBA F1 donors. Transgenic mice were identified by PCR with primers AGAAGACAGTGGAGGGAGCA from the synclein coding sequence and TCGTATAGGGTAGGCTTCAGG spanning the synuclein coding sequence and the HA tag. Two lines of transgenic mice were established. All of the reported work was done with a single line of mice (Tg13). Transgenic mice were backcrossed onto the C57BL/6J background. All experiments reported here used hemizygous transgenic mice. Striatal dopamine levels were measured as previously described^3^.

Clinical evaluations

Pole test was preformed essentially as described (13), briefly mice were placed head up onto a vertical wooden pole (50cm x 1cm diameter) placed in their home cage. The time taken for the mouse to turn and orient themselves head down was measured (turn), and the total time taken to turn and walk down to the base was measured (Total). Mice were trained on 2 separate occasions and then tested again after 7 days. Each mouse was given 5 attempts and the best 2 times were averaged.

Spontaneous activity measurements: Mice were placed in a clean transparent cylinder (height 16cm, diameter 13cm) in a quiet room. The mice were monitored for 3 mins and the number of rearings (both forelimbs off the ground) and the time grooming (seconds) measured.

Cell culture and exosome purification

The human SH-SY5Y neuroblastoma cell line (American Type Culture Collection) clones constitutively expressing full-length WT mouse or human S129D alpha-synuclein with a C-terminal haemagglutinin (HA) tag were cultured using standard conditions^4^. Murine dendritic cells harvested from bone marrow were cultured (3x10^6^ cells per well, 6 well/plate) in DMEM Glutamax (Gibco-BRL), 10% Foetal Calf Serum (FCS) and penicillin/streptomycin, Supplemented with 10ng/ml murine GM-CSF (MP Biomedicals). Cells were transfected after 4 days with 5µg of RVG-Lamp2b plasmid and 5µl of TransIT LT1 transfection reagent (Mirus Bio) as per manufacturer’s instructions. Cell culture medium was changed on Day 7 and cell culture supernatant harvested after 24 hours, centrifuged at 12,000g for 30 min to remove cell debris, then centrifuged again at 120,000g for 1 hour to pellet exosomes. Exosomes were resuspended in 0.1M ammonium acetate with a 27G needle. Foetal Calf Serum (FCS) used for exosome production was centrifuged at 120,000g for 60 minutes before preparation of medium.

Treatment of Cells with siRNAs

Cells were transfected using HiPerfect Transfection reagent (Qiagen, Hilden, Germany) with 100 nM siRNAs (Eurogentec) listed in Table 1, for 72 hours to determine their efficiency. To load siRNAs into exosomes 100 nM of siRNA and 3 µg of RVG-exosomes were electroporated (400v, 125 mA) in a 4mm cuvette using GenePulse Xcell electroporator (BioRad).

Exosome treatement of Mice.

The exosome distribution experiments were approved by The Swedish Local Board for Laboratory Animals and performed in accordance with the ethical permission. Other experiments were carried out in the animal unit, Royal Free Campus, University College London, London, UK according to procedures authorized by the UK Home Office. All animal experiments where designed to minimize the suffering and pain of the animals.

For the analysis of the in vivo brain distribution of RVG-exosomes the conditioned media from RVG-transfected DCs was filtered through 200 nm filter and the exosomes was incubated at room temperature for 15 minutes with 1 μM DiR (D12731, Invitrogen). The use of DiR labelling of exosomes for distribution studies has been described previously^5,6^. The exosomes were pellet by ultracentrifugation at 110 000g for 70 min. A washing step was then preformed by re-suspending the pellet in 25 ml 0.01M PBS and subsequent ultracentrifugation at 110 000g for another 70 min. The pelleted exosomes were re-suspended in 0.01M PBS. Particle analysis was performed with The NS500 nanoparticle analyzer (NanoSight, United Kingdom) to measure the size distribution of particles, which is based on the motion of nanometre-sized particles (Brownian motion)^7^. The number of particles and their movement were recorded for 5x60s and analyzed using the NS500 software. 1x1012 particles/ml of DiR labelled exosomes were injected in the tail vein of C57BL/6 female mice. 24 hours post injection the animals were sacrificed and the brains were carefully harvested and imaged for 2 seconds (excitation 710, emission 760) using an In Vivo Imaging System (IVIS) Spectrum (PerklinElmer, MA, USA). IVIS has previously been used to analyse the organ distribution of DiR-labelled exosomes^11^. The data was analyzed with the IVIS software (Living Image Software for IVIS®).

12-14 week-old normal C57BL/6 mice (males and females) and 20-22 week-old C57BL/6 transgenic mice (line 13, males and females) expressing the human S129D alpha-synuclein cDNA with a C-terminal HA tag under the PrP promoter were used for experiments. 150µg of alpha-synuclein siRNA was electroporated into 150µg of siRNA RVG-exosomes. RVG-exosomes containing siRNAs for in vivo experiments were centrifuged 120,000g for 1 hour and resuspended in 80µl of 5% glucose immediately before tail vein injection. Animals were sacrificed 3 or 7 days after injection.

Alpha-synuclein sequential extraction

Brain samples were sequentially extracted in high salt with sonication, (HS, 50 mmol/l Tris, 750 mmol/l NaCl, 5 mmol/l EDTA); HS/Triton X-100 (1% Triton X-100); and SDS/urea (8 mol/l urea, 2% SDS). In between each step insoluble material was pelleted by centrifugation at 60,000g and the pellets washed ^3^.

Western blotting

Cell samples were solubilised, separated on NuPAGE Novex 4%-12% Bis-Tris Gel (Invitrogen) and analysed by Western blot as previously described^3^ using the following primary antibodies; anti HA (1:5000, 71-5500, Invitrogen) ; anti alpha-synuclein (1:5000, Ab1903); anti beta-actin (1:30000, Ab6276) (all Abcam, Cambridge, England). Horseradish peroxidase–conjugated antimouse IgG secondary antibody (DAKO, Ely, England), was detected using ECL Western Blot Substrate (Pierce) and Hyperfilm ECL (GE Healthcare, Little Chalfont, England). Films were scanned and signals in the linear range were quantified using Image J and normalized to beta-actin levels.

Quantitative PCR.

Total RNA was harvested using the RNeasy kit (Qiagen) as per manufacturer's protocol. Reverse transcription (RT) was performed with qSCRIP Reverse Transcriptase kit (Primer Design, Southampton, England) as per manufacturer's instructions. qPCR experiments were performed on a StepOne^TM^ Real-Time PCR system (Applied Biosystems) using Precision qPCR Mastermix (Applied Biosystems). Values were calculated using the standard delta-delta Ct method.

Thioflavin S staining

Mice brains were fixed in 4% paraformaldehyde for 48 hours and cryoprotected in 30% sucrose . Coronal sections (20 μM) were cut with a frozen microtome (Leica) for histopathological assessment. Brain slices were mounted onto a slide and allowed to completely air dry. Slides were washed with 70%ethanol and 80% ethanol and incubated with thioflavin S solution (1% in 80% ethanol) for 15 minutes. Slides were sequentially washed with ethanol 80% and 70% ethanol, distilled water and mounted in aqueous mounting medium and analyzed with a Leica TCS-SL confocal microscope and images captured under identical conditions were analyzed with Image J.

Immunohistochemistry

For immunofluorescence slices (30µm) were washed with PBS and were treated in a blocking solution (0.1 M PBS containing 4% normal goat serum 0.04% Triton X-100) for 1 h. Slices were incubated overnight at room temperature with the primary antibodies: rat anti-TH (1:1000, MAB5280, Chemicon), monoclonal mouse anti-HA (1:1000, Invitrogen) Slices were washed three times with PBS and were then incubated with a secondary antibody of the appropriate species: Alexa 593 goat anti-mouse (1:200), Alexa 488 goat anti-rat (1:200) were obtained from Molecular Probes. All the samples were processed simultaneously to obtain comparable staining intensities.

Immunohistochemical analyses were performed on mid-sagittal sections (7µm) using the mouse on mouse immunodetection kit (Vector labs), anti HA antibody primary antibody (1/1000, Invitrogen), biotinylated anti mouse secondary antibody, followed by the avidin-biotin-peroxidase complex (ABC, Vector Labs). The antibodies were visualised using 3,3-diaminobenzidine (DAB) as the chromagen.

Statistical Analysis

Statistical analyses of the data were performed using SPSS, program 21.0, using the non-parametric Kruskall-Wallis and Mann-Whitney U test for in vitro experiments and parametric student T test for in vivo studies .

**SUPPLEMENTARY REFERENCES**

1. Al-Shawi R, Burke J, Jones CT, Simons JP, Bishop JO. A Mup promoter-thymidine kinase reporter gene shows relaxed tissue-specific expression and confers male sterility upon transgenic mice. Mol Cell Biol. 1988; 8: 4821-4828.

2. Borchelt DR, Davis J, Fischer M, Lee MK, Slunt HH, Ratovitsky T, Regard J, Copeland NG, Jenkins NA, Sisodia SS, Price DL. A vector for expressing foreign genes in the brains and hearts of transgenic mice. Genet Anal. 1996; 13: 159-163.

3. Rose S, Nomoto M, Jenner P. Quantification of dopamine and its metabolites by HPLC. Biochem Pharmacol. 1989; 38: 3677–3681.

4. Alvarez-Erviti L, Seow Y, Schapira AH, Gardiner C, Sargent IL, Wood MJ, et al. Lysosomal dysfunction increases exosome-mediated alpha-synuclein release and transmission. Neurobiol Dis*.* 2011; 42: 360-367.

5. Hood JL, San RS, Wickline SA. Exosomes released by melanoma cells prepare sentinel lymph nodes for tumor metastasis. Cancer research. 2011; 71: 3792-3801.

6. Ohno S, Takanashi M, Sudo K, Ueda S, Ishikawa A, Matsuyama N, *et al*. Systemically injected exosomes targeted to EGFR deliver antitumor microRNA to breast cancer cells. Mol Ther. 2013; 21: 185-191.

7. Dragovic RA, Gardiner C, Brooks AS, Tannetta DS, Ferguson DJ, Hole P, *et al*. Sizing and phenotyping of cellular vesicles using Nanoparticle Tracking Analysis. Nanomedicine. 2011; 7, 780-788.

**Supplementary Figure 1. In vitro alpha-synuclein down-regulation.** SH-SY5Y cells over-expressing mouse alpha-synuclein-HA were treated with 100 nM of three different siRNAs (siRNA 1, 2 or 3) using Hiperfect transfection reagent or 3 μg of RVG exosomes loaded with different siRNAs. alpha-synuclein mRNA (a) and protein down-regulation (b) after three days.

**Supplementary Figure 2. In vivo distribution of RVG-exosomes.** (a) C57bl/6 mice were injected with RVG-exosomes labelled with the fluorescent IRDye marker (n=3). 24h after intravenous injection the brains were analysed using the IVIS-system to quantitate the fluorescent IRDye signal in the whole brain. Typical scans of the brains from 2 mice injected with RVG-exosomes labelled with IRDye and 2 controls. Data was calculated for the whole brain and the signal compared to control injected mouse brains (n=3). (b) RVG-Exosomes were isolated from denditric cells culture and their size distribution measured by NTA, a typical profile is shown. Data normalised to control levels and expressed as mean ± SEM (n=3). Statistical analyses compared to control group, **<0.01

**Supplementary Figure 3. Evaluation of behaviour and dopamine level in transgenic mice.** (a) Time to orient down (t-turn) and total time to descend the pole (t-total) were measured for normal (n = 8) and Tg13 (n = 8) mice at 6 months of age (b) Spontaneous activity (rearing and time spent grooming) in normal (n = 8) and Tg13 (n = 8) mice at 9 months of age. (c) Dopamine levels in striatum of normal (n = 8) and Tg13 (n = 8) mice at 6 months of age. Statistical analyses compared to control group.

**Supplementary Figure 4. Evaluation of the alpha-synuclein down-regulation in other brain regions of Tg13 mice.** (a) Alpha-synuclein-HA protein levels were evaluated relative to actin in; brain stem (BS), thalamus (Th), olfactory lobe (OL), spinal cord (cord) and cerebellum (CBL) from Tg13 mice after 7 days siRNA3 RVG-exosome treatment and compared to untreated Tg13 mice. Data expressed as mean ± SEM (n=8). Statistical analyses compared to control group, *<0.05, ** <0.01. (b) Representative western blot of alpha-synuclein-HA and actin in brain stem, olfactory lobe, midbrain, thalamus, cerebellum and cord.

**Supplementary Figure 5. Evaluation of the S129D alpha-synuclein down-regulation in SH-SY5Y with control siRNA and siRNA3.**  SH-SY5Y cells over-expressing human S129D alpha-synuclein-HA were treated with 100 nM siRNA3 or control siRNA using Hiperfect transfection reagent for 72 hours. (a) Alpha-synuclein mRNA levels were quantified by qPCR relative to GAPDH and (b) human S129D alpha-synuclein (HA) and actin protein levels by Western blot. (c) Immunofluorescent analysis of S129D alpha-synuclein-HA expression in TH positive neurons, S129D alpha-synuclein-HA staining intensity was quantified using Image J (20 neurones analyzed per animal).
